# The gray boundaries of aberrant shortening of the cellular timekeepers’ edges

**DOI:** 10.1038/s44321-024-00122-1

**Published:** 2024-08-28

**Authors:** Guillermo Guenechea, Nestor W Meza

**Affiliations:** Centro de Investigaciones Energéticas Medioambientales y Tecnológicas and Centro de Investigación Biomédica en Red de Enfermedades Raras (CIEMAT/CIBERER), Advanced Therapies Unit, Instituto de Investigación Sanitaria Fundación Jiménez Díaz (IIS-FJD UAM), Madrid, Spain

**Keywords:** Genetics, Gene Therapy & Genetic Disease

## Abstract

N. Meza and G. Guenechea discuss novel genetic variants identified in telomere biology disorder dyskeratosis congenita (DC) and DC-like patients, as reported by Tummala and colleagues in this issue of *EMBO Mol Med*.

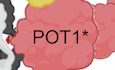

## Teaser

Telomeres are supramolecular structures that allow the DNA strand to fold back on itself and protect the linear chromosome end from being sensed as a double-strand DNA break. Telomeric conservation relies on mechanisms that replace terminal DNA sequences, and ensuring structural integrity. Telomere biology disorders (TBDs) are a heterogeneous group of low-prevalence pathologies defined by germline mutations in genes involved in telomere maintenance mechanisms (TMMs). TBDs manifest across a broad clinical spectrum, often with substantial phenotypic and genetic overlap among clinical entities. In this issue of *EMBO Molecular Medicine*, Tummala and collaborators present clinical and biological data from DC/DCL patients, that enhances the understanding of the natural history of these diseases. In addition, the description of novel TBD-associated genetic variants in *POT1* and *ZCCHC8* and of the new *POLA1* gene advances the understanding of the functional network of genes involved in TBD and highlights new pathogenic mechanisms.

**Figure 1:**
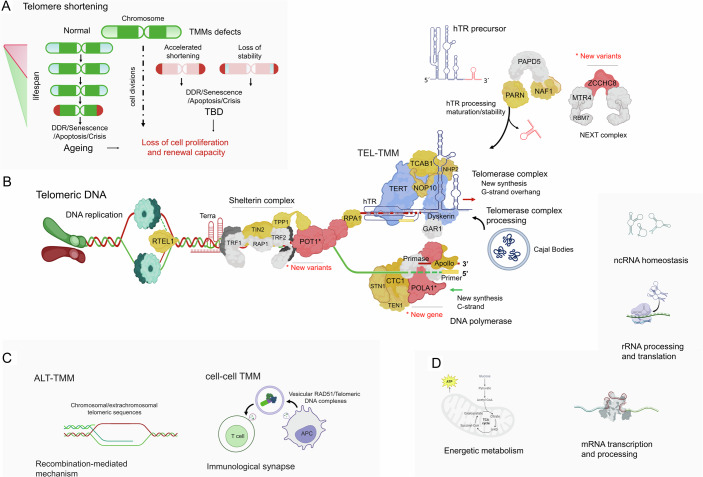
Overview of telomere maintenance mechanisms and their functional roles within and beyond telomeres. (**A**) Graphic diagram of telomere attrition in somatic cells during normal aging and TBDs. Normal telomere shortening occurs with each cell replication, but accelerated or sudden loss of telomeric sequences is caused by dysfunction in telomere elongation or stabilization factors, respectively. (**B**) An outline of the structure of the major components of telomeric termination. Representation of the telomerase, shelterin, CST complexes, and other factors involved in the maintenance of telomeric DNA. The telomerase complex includes hTR (human telomerase RNA component), TERT (telomerase reverse transcriptase), dyskerin, NOP10, NHP2, GAR1, and TCAB1. The shelterin complex is composed of TRF1, TRF2, TPP1, POT1, RAP1, and TIN2. The telomere capping (CST) complex consists of CTC1, STN1, and TEN1. POLA1 is a new X-linked genic component in the complex architecture of DC, and its function is related to protein-protein interactions with primase and shelterin subunit complexes. hTR regulators include NAF1, PARN, and ZCCHC8. Other accessory factors for telomere replication/protection and 3’ G-overhang regulation are RTEL1, RPA1, and Apollo. New variants described in Tummala et al (2024) appear in red (*POT1*, *POLA1*, and *ZCCHC8* genes). Yellow and blue colored components are associated with TBDs. The most frequently mutated genes are represented in blue. The factors in gray are products that have not been described as telomere disorder-causing genes. (**C**) The panel shows two additional mechanisms associated with telomere length maintenance. The recombination-dependent ALT mechanism is shown on the left of the panel, and the “altruistic” telomeric transfer and elongation by telomere/Rad51-containing extracellular vesicles between antigen-presenting cells and T lymphocytes in the immune synaptic space is depicted on the right of the panel. (**D**) The figure provides a concise summary of the general cellular processes in which the involvement of TMM components has been documented. Tummala et al (2024) suggest a role for ZCCHC8 in lncRNA transcription and on the anti-inflammatory/inflammatory balance. TBD Telomere Biology Disorder, DDR DNA Damage Repair, TMM Telomere Maintenance Mechanisms, DC Dyskeratosis Congenita, ALT Alternative Lengthening of Telomeres.

Telomeres are nucleoprotein protective caps at the ends of chromosomes. These supramolecular structures allow the DNA strand to fold back on itself and protect the linear chromosome end from being sensed as a double-strand DNA break (Chakravarti et al, 2021). Under normal circumstances, telomeres are part of a finely tuned biological system in which each time a cell divides (and the cell’s DNA is replicated), the telomere ends shorten. Eventually, much of the telomere sequence is lost, preventing the formation of protective loops and the cell being able to continue replicating, which results in cellular senescence (Fig. 1A). Therefore, telomeres protect chromosomes during all cell divisions of a normal lifespan, depending on their initial length and the rate at which they are shortened (Lansdorp, 2022). Germinal and early embryonic cells efficiently circumvent periodic telomeric shortening during cell division cycles. In addition, adult progenitor cells and certain types of lymphoid cells partially prevent physiological telomere attrition, thereby preserving the repopulating potential of proliferative tissues and the immune system to ensure protective long-term surveillance and immunological memory. The functional conservation of telomeres relies on mechanisms that facilitate the replenishment of telomeric sequences lost during DNA replication, concomitantly ensuring the structural integrity of telomeres (Kirwan and Dokal, 2009; Lansdorp, 2022; Revy et al, 2023). These telomere maintenance mechanisms (TMMs) involve the action of molecular machines controlled temporally and spatially, that are able to add new DNA (containing many repeats of the TTAGGG sequence) to the ends of chromosomes, which then counteract the shortening process that accompanies cell division.

The telomerase pathway (Fig. 1B) activates the typical TMM. The Telomerase TMM (TEL-TMM) utilizes the telomerase ribonucleoprotein that contains an RNA template for telomeric DNA synthesis. TEL-TMM is active in germline, and to a certain extent in stem cells, but not in mature somatic cells (Revy et al, 2023). Although most pathways used for telomere maintenance uses telomerase, in some circumstances the telomere length is maintained by a recombination-mediated mechanism called Alternative Lengthening of Telomeres (ALT) (Fig. 1C). ALT is an extremely rare TMM in the physiology context; however, a lower proportion of tumors (around 10%) activates the ALT recombination pathway between different sources of telomeric sequences (Kent and Clynes, 2021). Recently, a non-autonomous cell–cell TMM was described in the immune system context. This “altruistic” cell–cell TMM occurs at the immunological synapse, where antigen-presenting cells transfer RAD51/Telomeric DNA complexes to CD4^+^ T cells during synaptic contact (Fig. 1C). This vesicular traffic of Telomeric DNA complexes, together with telomerase, helps T cells to handle telomere attrition and evade cellular senescence (Lanna et al, 2022). As the cellular machinery capable of maintaining telomere integrity was dissected, the understanding of its differential impact on the telomere elongation, as well as on the replicative potential and senescence of different cell populations increased. In addition to their telomeric functions, the TMM components are involved in numerous cellular processes (extratelomeric functions) (Fig. 1D). The extensive network of telomeric and extratelomeric functions of the components of TMMs provides evidence of the complex relationship between alterations in the maintenance of human telomeres and a wide range of pathologies, cancer and aging.

Telomere biology disorders (TBDs) are a heterogeneous group of low-prevalence pathologies genetically defined by germline mutations in genes involved in TMMs. The complexity of TBDs is underscored by the diversity of TMM components that may be involved in the observed molecular and cellular damage. TBDs are caused by the loss of their classical telomeric functions and their possible involvement in other cellular functions. A common feature of patients with TBDs is the presence of very short telomeres for their age, which is a predictor of early-age injury of tissues and organs with high rates of proliferation and renewal, such as the epithelium and hematopoietic system (Fig. 1A). Short telomeres expose these patients to a high risk of a wide range of health issues, including bone marrow failure, cancer, arteriovenous malformations, pulmonary fibrosis, liver disease, stenosis of the urethra, esophagus, or lacrimal ducts and other unrelated medical complications. Therefore, TBDs manifest across a broad clinical spectrum, often with substantial phenotypic and genetic overlap among various clinical entities. Significant clinical variability can be observed even among family members carrying identical germline mutations (Lansdorp, 2022; Kam et al, 2021). These factors, combined with the inheritance patterns of genetic mutations - autosomal dominant, autosomal recessive, or X-linked recessive - pose significant challenges to early diagnosis and treatment efforts. The archetypal TBD is dyskeratosis congenita (DC), which is characterized by dermatologic manifestations and bone marrow failure that typically present during childhood or adolescence. Høyeraal–Hreidarsson syndrome, Revesz syndrome and Coats plus are the most severe forms of early-onset TBDs, in which critically ill patients experience the failure of multiple organ systems within the first decade of life. In adulthood, the disease typically manifests with pulmonary fibrosis or bone marrow failure as the primary clinical presentation. In families carrying certain mutations, an inherited pattern of genetic anticipation has been demonstrated, where the age of becoming symptomatic is progressively reduced with each generation, and the clinical manifestations of the disease worsen in successive generations. Additionally, some individuals may experience very mild symptoms or remain asymptomatic, resulting in a life expectancy comparable to that of individuals not carrying TBD-associated mutations (Revy et al, 2023; Kam et al, 2021; Niewisch et al, 2022).

In recent years, there has been a concerted and systematic effort to elucidate the genetic landscape of these complex disorders by evaluating modes of inheritance and their relationship to clinical progression. Comprehensive longitudinal studies enriched with extensive genetic, clinical, and epidemiological data are generating robust resources to enhance management strategies (Niewisch et al, 2022; Revy et al, 2023). In this issue of *EMBO Molecular Medicine*, Tummala and collaborators present the results of an extensive effort to conduct a thorough clinical and genetic investigation, rigorously categorizing a large collection of clinically diagnosed cases of DC, as well as cases exhibiting features resembling DC, referred to in the manuscript as ‘DC-like’ (DCL). In addition, their work comprehensively defines new genetic variants and identifies a new gene involved in the alteration of TMMs (Tummala et al, 2024). The study describes the molecular involvement of newly identified genetic variants in *POT1* and *ZCCHC8* and the novel X-linked *POLA1* gene in critical telomeric maintenance mechanisms (Fig. 1), and the impact on various extratelomeric functions. This expands the intricate network implicated in the underlying pathology.

Several elegant studies performed by Tummala et al, provide valuable information on how the disruption of the interaction of *POLA1* and *POT1* with the CST complex contributes to molecular dysfunction in DNA synthesis from the 5’ end of DNA, which gives a plausible explanation for the phenotypes observed in novel *POLA1* and *POT1* DC/DCL cases (Fig. 1B). The description of the role of *ZCCHC8* in transcript degradation, particularly the regulation of snoRNAs encoded within the lncRNAs *GAS5* and *LITE1* RNAs, suggests a broader impact of *ZCCHC8* variants on nuclear RNA homeostasis (Fig. 1B). Moreover, Tummala and collaborators propose that *PAPD5* inhibitors could help to restore hematopoietic potential in those patients and that specific targeting of *GAS5* regulation could be a possible new treatment for these patients.

Notably, the authors mention several genes involved in DC in their introduction but omit *POT1*, which is primarily associated with Coats plus syndrome, even though they describe the molecular characterization of pathogenic POT1 variants in DC/DCL cases. This omission highlights the ambiguous boundaries between different TBDs and the challenges in the genetic categorization of this spectrum of diseases, which become increasingly complex as new genes and extratelomeric functions are identified. The functional network of genes involved in TBD underscores the need for continuous efforts to achieve genetic, molecular, and clinical consensus. Such global collaboration is essential for strengthening early diagnosis, providing effective genetic counseling, improving treatment, and advancing the development of innovative therapies to better manage and treat these complex disorders.

Finally, as the authors point out in their study, the collection of clinical and biological data from DC/DCL patients, along with the identification of DC cases within the group of patients with DCL using rigorous evaluation criteria, undoubtedly enhances the understanding of the natural history of these diseases. This approach facilitates the discovery of disease-associated genetic variants and allows for accurate evaluation of therapeutic approaches. Molecular characterization of new variants and implicated genes is advancing the understanding of the pathogenic mechanisms of TBD and will provide potential therapeutic targets.
